# Divergence in Cigarette Discontinuation Rates by Use of Electronic Nicotine Delivery Systems (ENDS): Longitudinal Findings From the United States PATH Study Waves 1–6

**DOI:** 10.1093/ntr/ntae027

**Published:** 2024-04-03

**Authors:** Karin A Kasza, Zhiqun Tang, Young Sik Seo, Adam F Benson, MeLisa R Creamer, Kathryn C Edwards, Colm Everard, Joanne T Chang, Yu-Ching Cheng, Babita Das, Olusola Oniyide, Nicole A Tashakkori, Anna-Sophie Weidner, Haijun Xiao, Cassandra Stanton, Heather L Kimmel, Wilson Compton, Andrew Hyland

**Affiliations:** Department of Health Behavior, Roswell Park Comprehensive Cancer Center, Buffalo, NY, USA; Behavioral Health and Health Policy, Westat, Rockville, MD, USA; Department of Health Behavior, Roswell Park Comprehensive Cancer Center, Buffalo, NY, USA; Center for Tobacco Products, US Food and Drug Administration, Silver Spring, MD, USA; National Institute on Drug Abuse, National Institutes of Health, Bethesda, MD, USA; Behavioral Health and Health Policy, Westat, Rockville, MD, USA; National Institute on Drug Abuse, National Institutes of Health, Bethesda, MD, USA; Center for Tobacco Products, US Food and Drug Administration, Silver Spring, MD, USA; Center for Tobacco Products, US Food and Drug Administration, Silver Spring, MD, USA; Center for Tobacco Products, US Food and Drug Administration, Silver Spring, MD, USA; Center for Tobacco Products, US Food and Drug Administration, Silver Spring, MD, USA; Center for Tobacco Products, US Food and Drug Administration, Silver Spring, MD, USA; Center for Tobacco Products, US Food and Drug Administration, Silver Spring, MD, USA; Center for Tobacco Products, US Food and Drug Administration, Silver Spring, MD, USA; Behavioral Health and Health Policy, Westat, Rockville, MD, USA; National Institute on Drug Abuse, National Institutes of Health, Bethesda, MD, USA; National Institute on Drug Abuse, National Institutes of Health, Bethesda, MD, USA; Department of Health Behavior, Roswell Park Comprehensive Cancer Center, Buffalo, NY, USA

## Abstract

**Introduction:**

We compare real-world trends in population-level cigarette discontinuation rates among adults (ages ≥21) who smoked cigarettes, by electronic nicotine delivery systems (ENDS) use.

**Aims and Methods:**

U.S nationally representative data from adults in the Population Assessment of Tobacco and Health (PATH) Study (2013/14-2021, Waves 1–6) who smoked cigarettes in the past 30 days (P30D) were analyzed (*n* = 13 640). The exposure was P30D ENDS use. The outcome was P30D cigarette discontinuation at biennial follow-up. Weighted trend analyses were conducted to test for differences in cigarette discontinuation trends by ENDS use.

**Results:**

Between 2013/14 and 2015/16, cigarette discontinuation rates were both 16% for those who used ENDS and for those who did not; between 2018/19 and 2021, rates were ~30% for those who used ENDS and ~20% for those who did not; the time by ENDS use interaction was significant.

**Conclusions:**

The relationship between adults’ ENDS use and cigarette discontinuation in the context of an expanded ENDS marketplace, new tobacco regulatory actions, and COVID-19 differs from the relationship in earlier years.

**Implications:**

It is important for public health decisions to be informed by research based on the contemporary ENDS marketplace and circumstances.

## Introduction

Electronic nicotine delivery systems (ENDS) first emerged on the U.S. market in 2007 resembling conventional cigarettes and using fixed low-voltage batteries but have since become available with customizable battery voltage and wattage.^1^ Beginning in 2016, e-liquids containing nicotine salt formulations became widely available, which are lower in pH than free-base formulations, allowing manufacturers to increase nicotine concentration while avoiding harshness and bitterness.^1^

Past population-level research has reported conflicting findings on whether ENDS use helps people who smoke combustible cigarettes to quit smoking.^2–7^ Some research suggests improved cigarette quitting-related outcomes with ENDS use,^2–4^ while other research suggests ENDS use is associated with lower rates of cigarette quitting.^5–7^ Inconsistent findings may be due in part to differences in the samples and measures considered, differences in analytic approaches used, and/or may be because of the rapidly changing product environment or differing policy contexts. We hypothesized that at the population level, cigarette discontinuation rates increased in recent years more among those who used ENDS than among those who did not use ENDS given the expansion of the ENDS marketplace. This descriptive paper examines differences in real-world trends in population-level cigarette discontinuation rates across 2013/14–2021, comparing U.S. adults who smoked combustible cigarettes and used ENDS with U.S. adults who smoked combustible cigarettes and did *not* use ENDS (regardless of other tobacco product use).

## Methods

### Participants

Data were analyzed from adults aged ≥21 years who participated in the Population Assessment of Tobacco and Health (PATH) Study, a nationally representative longitudinal study of youth and adults in the United States.^8^ The age cutoff was selected to correspond to the minimum age for legal sale of tobacco products in the context of “Tobacco 21,” which became federal law during our study period^9^ and which was state law in several states prior to becoming federal law.^10^ PATH Study data were collected using audio computer-assisted self-interviews conducted in English or Spanish from September 2013 to December 2014 (Wave [W]1), October 2014 to October 2015 (W2), October 2015 to October 2016 (W3), December 2016 to January 2018 (W4), and December 2018 to November 2019 (W5). W6 data collection took place from March 2021 to November 2021; because of COVID-19 pandemic restrictions in effect in 2021, W6 data were collected using both audio computer-assisted self-interviews and telephone interviews. In-person data collection was prioritized over telephone data collection where it was permitted by local jurisdictions and participant comfort.^8^

The PATH Study employed a stratified address-based, area-probability sampling design at W1 that oversampled people who use tobacco, those aged 18–24 years, and African Americans. An in-person screener was used to randomly select individuals from households for participation in the study. Study participants recruited at W1 formed the W1 cohort. At W4, a probability replenishment sample was selected from the United States civilian noninstitutionalized population (CNP) at the time of W4, including persons who were not in the CNP at the time of W1 (such as people who recently immigrated or returned from the military). The within-household sampling procedures mirrored those used at W1, with sampling rates varying by age, race, and tobacco-use status. Members of the W1 cohort who were in the CNP at the time of W4 were combined with the W4 replenishment sample to form the W4 cohort. Further details are available at https://doi.org/10.3886/Series606.^8^

The PATH Study is conducted by Westat and approved by the Westat Institutional Review Board. All adult participants aged ≥18 years provided informed consent. The overall weighted response rate for adults in the W1 cohort was 74.0% at W1, 83.2% at W2, 78.4% at W3, 73.5% at W4, 69.4% at W5, and 57.5% at W6. The overall weighted response rate for adults in the W4 cohort was 73.5% for the continuing sample and 68.0% for the replenishment sample at W4, 88.0% at W5, and 73.5% at W6.^8^ Further details regarding the PATH Study design and methods^11–13^ and demographic and tobacco use distributions^14^ are published elsewhere. Details on interviewing procedures, questionnaires, sampling, weighting, response rates, and accessing PATH Study Restricted Use Files are available at https://doi.org/10.3886/Series606.^8^ This manuscript follows the STROBE reporting guideline for cohort studies (https://www.equator-network.org/reporting-guidelines/strobe/).

We conducted analyses among adults aged ≥21 years who smoked combustible cigarettes at the baseline wave of any biennial wave pair (ie, those aged 21 or older who smoked cigarettes in the past 30 days at baseline, had ENDS use data at baseline and had cigarette discontinuation data at follow-up, *n* = 13 640 individuals). Estimates were weighted to adjust for the PATH Study’s complex study design characteristics (eg, oversampling) and attrition, making them representative of the resident adult population of the United States at the time of data collection, who were also in the CNP.^8^ Variances were computed using the balanced repeated replication (BRR) method^15^ with Fay’s adjustment set to 0.3 to increase estimate stability.^16^ The Wilson method was used to calculate 95% confidence intervals (CIs).

### Measures

Respondents were asked about their demographic characteristics and, at each interview, whether they smoked cigarettes in the past 30 days and whether they used ENDS in the past 30 days. Table 1 shows the predictor and outcome measures used in analyses, and the demographic variables used to describe the population.

**Table 1.** Measures

Measures^a^	Categorizations^b^	Questions used in categorizations
**Predictor measures (two approaches: assessed at baseline wave of each wave pair; assessed at follow-up wave of each wave pair)**		
ENDS use at baseline	(1) P30D ENDS use: use ENDS in P30D at baseline. (2) No P30D ENDS use: did not use ENDS in P30D at baseline.	“In the past 30 days, have you used an electronic nicotine product, even one or two times? (Electronic nicotine products include e-cigarettes, vape pens, personal vaporizers and mods, e-cigars, e-pipes, e-hookahs, and hookah pens.)”^c^
ENDS use at follow-up	(1) P30D ENDS use: use ENDS in P30D at follow-up. (2) No P30D ENDS use: did not use ENDS in P30D at follow-up.	
**Outcome measure (assessed at follow-up wave of each wave pair)**		
Cigarette discontinuation at follow-up	(1) Discontinued cigarette smoking: did not smoke cigarettes in P30D at follow-up. (2) Did not discontinue cigarette smoking: smoked cigarettes in P30D at follow-up.	“In the past 30 days, have you smoked a cigarette, even one or two puffs?”
**Other measures (assessed at baseline wave of each wave pair)**		
Cigarette smoking status	P30D cigarette smoking: smoked cigarettes in P30D	“In the past 30 days, have you smoked a cigarette, even one or two puffs?”
Biological sex	Male, female	“What is your sex?”
Sexual orientation	Gay or lesbian, straight, bisexual, something else, and unknown	“Do you consider yourself to be . . . Straight; Lesbian or gay; Bisexual; Something else”
Race/ethnicity	Non-Hispanic White, non-Hispanic Black, non-Hispanic other racial group including multi-racial groups, Hispanic, and unknown	“Are you Hispanic, [Latino/Latina/Latino or Latina], or of Spanish origin? Choose all that apply.” “What is your race? Choose all that apply.”
Age	Continuous measure	“What is your age? “
Educational attainment	Less than high school/general equivalency diploma, high school graduate, some college/associate’s degree, Bachelor’s degree or more, and unknown	“What is the highest grade or level of school you have completed?”
Annual household income	<$25 000, $25 000–$74 999, $75 000+, and unknown	“Which of [category] best describes your total household income in the past 12 months?”

P30D = past 30 days.

^a^Missing data on age, sex, race/ethnicity, and educational attainment were imputed at wave 1 (and sex was also imputed at Wave 4) as described in the PATH Study Restricted Use Files User Guide (http://doi.org/10.3886/Series606]).

^b^For sexual orientation, race/ethnicity, education, and annual household income, when not imputed by the PATH Study, we included any missing as a separate “Unknown” category.

^c^In Wave 1 and Wave 2, e-cigarettes were the only product named in the question.

### Statistical Analyses

We evaluated cigarette discontinuation rates at follow-up using wave pairs to reflect approximately biennial intervals (ie, W1–W3, W2–W4, W4–W5, W5–W6, where W1, W2, W4, W5 serve as baseline waves to follow-up W3, W4, W5, W6, respectively). This enabled us to maximize our sample size, include data from the most recently available wave at the time of analysis, and have generally comparable timespans among wave pairs since the PATH Study switched from annual data collection to biennial data collection for adults between W4 and W5.^8^

Cigarette discontinuation rates were stratified by ENDS use at baseline in the first approach and were stratified by ENDS use at follow-up in the second approach. We used these two approaches for the assessment of ENDS use because each approach has different advantages. ENDS use assessed at baseline ensures that the ENDS use preceded the cigarette discontinuation outcome (although this approach misses ENDS use that occurred in the approximately 2 years after baseline and before/at follow-up). ENDS use assessed at follow-up ensures that the ENDS use occurred nearest to the cigarette discontinuation outcome (although this approach does not distinguish between whether ENDS use preceded or followed cigarette discontinuation).

Estimates were weighted using the single-wave or all-waves weights as appropriate and available for longitudinal analyses as described in detail by Kasza et al. (2022),^17^ including full-sample and 100 replicate weights (ie, W1–W3 estimates were weighted using W3 all-waves weights for the W1 cohort, W2–W4 estimates were weighted using W4 all-waves weights for the W1 cohort, W4–W5 estimates were weighted using W5 single-wave weights for the W4 cohort, and W5–W6 estimates were weighted using W6 all-waves weights for the W4 cohort); see also Figure and Supplementary Figure footnotes. Because of interview protocol differences in W6, where the mode of data collection depended on where and when in-person visits were permitted as well as participant comfort, we generated W6 estimates among all respondents, and separately among those interviewed in-person and among those interviewed by telephone as a sensitivity analysis. This sensitivity analysis is important as protocol differences may affect the demographic composition of people who responded via telephone versus in-person, the interview setting, as well as the answers respondents provide.^18^ For each wave pair, Rao–Scott Design-based F tests were used to evaluate differences in cigarette discontinuation rates between those who did and did not use ENDS (weighted and unadjusted to indicate what the rates look like in the population over the course of the study period).

We used generalized estimating equations (GEEs) logistic regression analyses to test the interactions between ENDS use and linear trends and between ENDS use and nonlinear trends (using a quadratic term and using a categorical variable comparing the most recent two wave pairs and comparing the first and last wave pairs) in cigarette discontinuation rates across W1–W6. We evaluated trends stratified by ENDS use and estimated odds ratios (ORs). Analyses were weighted using the W6 all-waves weights for the W1 cohort and were conducted unadjusted to reflect how the trends appear in the population, consistent with the population-level depiction from the biennial wave pair estimates. We also ran sensitivity analyses for biennial wave pair estimates using the subset of respondents present in all six waves and the W1 cohort.

GEE allows for inclusion of multiple wave pair observations in a single analysis while statistically controlling for interdependence among observations contributed by the same individuals.^19,20^ We specified the unstructured covariance and within-person correlation matrices and the binomial distribution of the dependent variable using the logit link function. Analyses were run on the W1–W6 Restricted Use Files (available at https://doi.org/10.3886/ICPSR36231).

## Results

The composition of the population of those who smoked cigarettes aged ≥21 years in this study (which includes repeated observations from individuals and is weighted to the 2013/14 [“Wave 1” cohort]) was as follows: 54.0% were male (95% CI: 52.9% to 55.1%); 2.6% identified as gay or lesbian (95% CI: 2.2% to 3.1%); 4.3% identified as bisexual (95% CI: 3.8% to 4.8%); 90.7% identified as heterosexual/straight (95% CI: 90.0% to 91.4%); 1.3% identified as other sexual orientation (95% CI: 1.1% to 1.5%); 1.1% identified as unknown sexual orientation (95% CI: 1.0% to 1.4%); 64.6% were non-Hispanic White (95% CI: 63.2% to 65.9%); 15.1% were non-Hispanic Black (95% CI: 14.2% to 16.0%); 5.5% were non-Hispanic other racial group including multi-racial groups (95% CI: 5.0% to 6.1%); 13.5% were Hispanic (95% CI:12.6% to 14.3%); 1.4% were unknown race/ethnicity (95% CI: 1.1% to 1.8%); 25.5% had less than high school/general equivalency diploma (95% CI: 24.5% to 26.5%); 27.3% were high school graduates without further education (95% CI: 26.2% to 28.5%); 33.4% had some college/associate’s degree (95% CI: 32.2% to 34.6%); 13.4% had a Bachelor’s degree or more education (95% CI: 12.6% to 14.4%); 0.4% had unknown educational attainment (95% CI: .3%to 0.5%); 44.6% had income <$25 000 (95% CI: 43.1% to 46.0%); 35.1% had income $25 000–$74 999 (95% CI: 34.0% to 36.1%); 14.7% had income $75 000 + (95% CI: 13.6% to 15.9%); 5.7% had unknown income (95% CI: 5.2% to 6.3%); and the median age was 41 years (range 21–90 years).

The distributions of the following sociodemographic characteristics differed between those who used ENDS and those who did not use ENDS at baseline (Rao–Scott Design-based F tests based on individuals, *n* = 7285 across the four wave pairs, *p* < .001): sexual orientation, with those who identified as heterosexual/straight being over-represented among those who did not use ENDS; race/ethnicity, with those who were non-Hispanic Black, Hispanic, or unknown race/ethnicity being over-represented among those who did not use ENDS; education, with those who had less than a high school/general equivalency diploma being over-represented among those who did not use ENDS; and age, with those who were older being over-represented among those who did not use ENDS (mean age = 41.0, brr SE = .3 for those who did not use ENDS vs. mean age = 35.5, brr SE = .3 for those who did use ENDS).

### Rates of Discontinuing Cigarette Smoking Between 2013/2014 and 2021, by ENDS Use at Baseline

The Figure shows cigarette discontinuation rates stratified by P30D ENDS use at baseline. Estimates for 2018/19–2021 (W5–W6) are presented among the full analytic sample for the Wave 4 cohort and are presented separately among those interviewed in-person in W6 and among those interviewed by telephone in W6 as sensitivity analyses. Cigarette discontinuation rates were higher among those interviewed by telephone than those interviewed in-person in W6, which was the case both for those who used ENDS and for those who did not use ENDS. Exploratory analyses indicated that there were differences in the demographic composition of those interviewed by telephone versus in-person in W6 (data not shown). However, because individuals in the PATH Study were selected with the use of a probability sample, the weighted cigarette discontinuation rates among the full analytic sample at W6 are statistically valid estimates for the adult U.S. resident population in 2021 (https://doi.org/10.3886/Series606.^8^)

**Figure 1.**
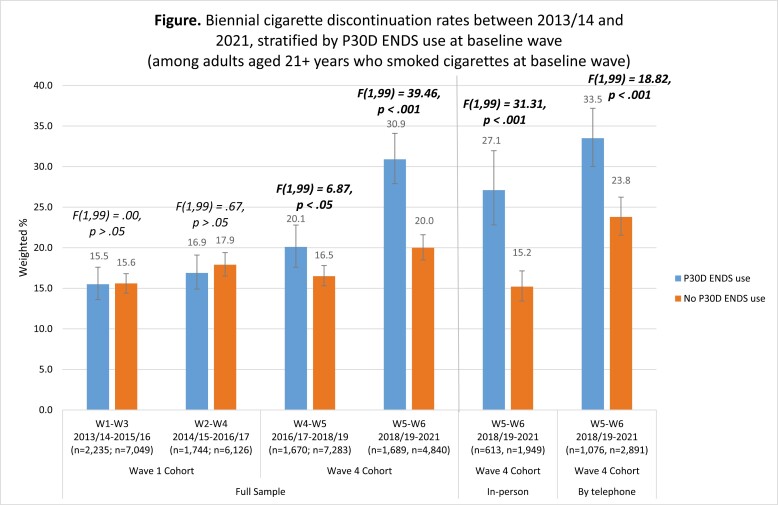
Cigarette discontinuation was defined as P30D smoking at baseline wave and no P30D smoking at follow-up wave for each biennial wave pair; ENDS use was defined as P30D ENDS use at baseline vs. no P30D ENDS use at baseline; analyses were weighted using the weights appropriate and available for each biennial wave pair as described in detail by Kasza et al. (2022),^17^ including full-sample and 100 replicate weights (ie, W1–W3 estimates were weighted using W3 all-waves weights for the W1 cohort, W2–W4 estimates were weighted using W4 all-waves weights for the W1 cohort, W4–W5 estimates were weighted using W5 single-wave weights for the W4 cohort, and W5–W6 estimates were weighted using W6 all-waves weights for the W4 cohort) such that all those who were eligible to participate in any interview pair were included in analyses and estimates represent cigarette discontinuation rates in the population at the time of the follow-up interview for those who were in the CNP at the time of W1 or W4. Sensitivity analyses using only the subset of respondents present in all five or six waves from the W1 cohort depending on which wave pair was involved (ie, using the W5 all-waves weights for the W1 cohort to evaluate the W4–W5 wave pair, and using the W6 all-waves weights for the W1 cohort to evaluate the W5–W6 wave pair) yielded findings consistent with those reported here.

Cigarette discontinuation rates were statistically indistinguishable between those who did and did not use ENDS until 2016/17 (Figure 1). Between 2016/17 and 2018/19 (W4 and W5), those who used ENDS had a higher rate of discontinuing cigarette smoking at follow-up than those who did not use ENDS (20.1%, 95% CI: 17.6% to 22.8% vs. 16.5%, 95% CI: 15.3% to 17.8%; F [1,99] = 6.87, *p* < .05). Between 2018/19 and 2021 (W5 and W6), those who used ENDS had an even higher rate of discontinuing cigarette smoking at follow-up than those who did not use ENDS (30.9%, 95% CI: 27.9% to 34.1% vs. 20.0%, 95% CI: 18.5% to 21.6%; *F*[1,99] = 39.46, *p* < .001).

Table 2 shows results from GEE trend analysis interactions between the various time trend terms and P30D ENDS use status at baseline, as well as P30D ENDS use-stratified trend results. Across 2013/14–2021 (W1–W6), there was a significant interaction between linear time × ENDS use and cigarette discontinuation rates (*p* < .001; OR = 1.19, 95% CI: 1.08 to 1.30) such that those who used ENDS experienced a steeper monotonic increase in cigarette discontinuation rates across the study period (OR = 1.35, 95% CI: 1.25 to 1.46) than did those who did not use ENDS (OR = 1.15, 95% CI: 1.11 to 1.20). There were also significant interactions between nonlinear time × ENDS use and cigarette discontinuation rates, with time as a categorical variable (*p* = .017; OR = 1.33, 95% CI: 1.05 to 1.68), when comparing the most recent wave pairs [2018/19–2021 vs. 2016/17–2018/19]; (*p* = .001; OR = 1.59, 95% CI: 1.21 to 2.09) and when comparing the first and last wave pairs [2018/19–2021 vs. 2013/14–2015/16]) such that there were greater increases in cigarette discontinuation rates for those who used ENDS than for those who did not use ENDS between the most recent wave pairs (OR = 1.69, 95% CI: 1.36 to 2.12 for those who used ENDS; OR = 1.30, 95% CI: 1.12 to 1.50 for those who did not use ENDS) and between the first and last wave pairs (OR = 2.42, 95% CI: 1.90 to 3.09 for those who used ENDS; OR = 1.57, 95% CI: 1.40 to 1.77 for those who did not use ENDS).

**Table 2.** Trends in Biennial Cigarette Discontinuation Rates Between 2013/14 and 2021 (W1 and W6), Stratified by P30D ENDS use at Baseline

	2013/14–2021 (W1–W6)*			
	Interactions		Stratified	
ENDS use at baseline	OR (95% CI)	*p* value	OR (95% CI)	*p* value
**Linear time term^#^**				
P30D ENDS use	1.19 (1.08–1.30)	<.001	1.35 (1.25–1.46)	<.001
No P30D ENDS use			1.15 (1.11–1.20)	<.001
**Nonlinear (quadratic) time term**				
P30D ENDS use	1.07 (1.00–1.16)	.066	1.10 (1.03–1.19)	.009
No P30D ENDS use			1.03 (0.99–1.07)	.195
**Nonlinear (categorical) time term: 2018/19–2021 vs. 2016/17–2018/19 (W5–W6 vs. W4–W5)**				
P30D ENDS use	1.33 (1.05–1.68)	.017	1.69 (1.36-2.12)	<.001
No P30D ENDS use			1.30 (1.12-1.50)	0.001
**Nonlinear (categorical) time term: 2018/19–2021 vs. 2013/14–2015/16 (W5–W6 vs. W1–W3)**				
P30D ENDS use	1.59 (1.21–2.09)	.001	2.42 (1.90-3.09)	<.001
No P30D ENDS use			1.57 (1.40-1.77)	<.001

W = wave; P30D = past 30-day; 95% CI = 95% confidence interval.

^*^There were protocol differences between W6 and W1–W5. P30D ENDS use: the number of observations = 4805 and the number of individuals = 3221; no P30D ENDS use: the number of observations = 16 627 and the number of individuals = 6515; analyses were weighted using the W6 all-waves weights for the W1 cohort.

^#^Estimates generated from the linear model (when the nonlinear [quadratic] time by ENDS use interaction term was nonsignificant, the linear models were also run and estimates from the linear models were presented here).

### Rates of Discontinuing Cigarette Smoking Between 2013/2014 and 2021, by ENDS Use at Follow-Up

Supplementary Figure shows cigarette discontinuation rates stratified by ENDS use at follow-up. As with the main Figure, estimates for 2018/19–2021 (W5–W6) are presented among the full analytic sample and are presented separately among those interviewed in-person in W6 and among those interviewed by telephone in W6 as sensitivity analyses. Cigarette discontinuation rates were statistically indistinguishable until 2018/19; in 2018/19–2021 (W5–W6), those who used ENDS had a higher rate of discontinuing cigarette smoking at follow-up than those who did not use ENDS (29.7%, 95% CI: 26.9% to 32.7% vs. 21.0%, 95% CI: 19.5% to 22.6%; F [1,99] = 28.96, *p* < .001).

Supplementary Table shows results from GEE trend analysis interactions between the various time trend terms and P30D ENDS use status at follow-up, as well as P30D ENDS use-stratified trend results. Across 2013/14–2021 (W1–W6), there was a significant interaction between quadratic time × ENDS use and cigarette discontinuation rates (*p* = .001; OR = 1.16, 95% CI: 1.06 to 1.26), such that those who used ENDS experienced a steep increase in cigarette discontinuation rates at the end of the study period (OR = 1.19, 95% CI: 1.09 to 1.29), and those who did not use ENDS did not experience such an increase (OR = 1.03, 95% CI: 0.99 to 1.07). Findings for interactions between nonlinear time × ENDS use and cigarette discontinuation rates were consistent with those reported previously when ENDS use status was assessed at baseline.

## Discussion

Nationally representative PATH Study data show that between 2013/14 and 2015/16 (W1 and W3), rates of discontinuing cigarette smoking among adults in the United States population were statistically indistinguishable between those who used ENDS and those who did not use ENDS (15.5% vs. 15.6%), while between 2018/19 and 2021 (W5 and W6), rates of discontinuing cigarette smoking were significantly higher for those who used ENDS than for those who did not use ENDS (30.9% vs. 20.0%; time by ENDS use interaction was statistically significant). These findings were generally consistent when ENDS use was assessed at baseline (which reflects dual use of ENDS and cigarettes prior to assessment of cigarette discontinuation) and when ENDS use was assessed at follow-up (which reflects ENDS use assessed alongside assessment of cigarette discontinuation), though cigarette discontinuation rates between people who did and did not use ENDS appeared to diverge sooner when ENDS use was assessed at baseline than when ENDS use was assessed at follow-up.

Our full study period spanned a time in the United States when the ENDS marketplace was expanding; salt-based nicotine formulations gained market share in 2016^1^ and ENDS products became available with increased nicotine yields over time,^21^ prevalence of ENDS use and frequent ENDS use was increasing,^22^ and various tobacco control actions were taken at state and federal levels. For example, several statewide Tobacco 21 laws were enacted,^10^ federal-level Tobacco 21 became effective in December 2019,^9^ some states and localities imposed ENDS flavor restrictions,^23–27^ and federal-level ENDS enforcement priorities became effective in February 2020.^28^ That is, much tobacco control activity took place between 2018/2019 and 2021, which is when we observed the large divergence in cigarette discontinuation rates between those who used ENDS and those who did not.

Additionally, the COVID-19 pandemic began in the United States in Spring 2020, at which time PATH Study data collection protocols changed, including switching from in-person to telephone interviewing, and in 2021, to both in-person and telephone interviewing.^8^ We observed differences in the 2018/19–2021 cigarette discontinuation rates between those interviewed by telephone and those interviewed in-person. Further, we observed differences in ENDS use, sex, income, race/ethnicity, and educational attainment between those interviewed by telephone and those interviewed in-person (results not reported). Overall, comparisons between 2021 and earlier years should be interpreted with caution. Even estimates among those interviewed in-person in 2021 may not be directly comparable to estimates from those interviewed in-person in previous years because of the protocol changes in 2021. While we observed some differences in sample characteristics and behaviors by interview mode, sensitivity analyses in which we compared the 2018/19–2021 cigarette discontinuation rates between those who used ENDS and those who did not by interview mode yielded findings consistent with the overall conclusions.

Other circumstances beyond changes in ENDS product characteristics, other market changes, changes in the policy environment, or the COVID-19 pandemic may also have occurred during our study period that could contribute to explaining why the relationship between ENDS use and cigarette discontinuation rates changed across 2013/14–2021. For example, it is possible that there have been changes in the characteristics of people who smoke cigarettes and use ENDS, interest in quitting smoking, reasons for ENDS use among those who smoke cigarettes, and/or use of other nicotine products among those who smoke cigarettes, though we are not aware of empirical evaluations of these possibilities.

Research has shown that daily use of ENDS is associated with cigarette discontinuation in the population^29–31^ and it is possible that ENDS use frequency increased over the course of our study period, perhaps because of changes in ENDS product features, which could contribute to explaining our findings. It is also notable that the latest Cochrane review of findings from clinical trials now concludes “with high certainty” that using ENDS with nicotine increases cigarette quit rates compared to using nicotine replacement therapy.^32^ Further research can investigate both potential explanations for our initial overall population findings here, as well as ENDS use initiation rates/cigarette discontinuation rates among young people across the same time period and beyond.

### Limitations

Limitations of this study include that we did not track ENDS use during the intervals *between* baseline and follow-up waves. Rather, in one approach, we evaluated ENDS use at baseline which was at the same time that we ascertained the sample of people who smoked cigarettes and in another approach, we evaluated ENDS use at follow-up which was at the same time that we ascertained the cigarette discontinuation outcome. The first approach may exclude people who used ENDS and discontinued cigarette smoking before baseline, and the second approach does not distinguish between whether ENDS use preceded or followed cigarette discontinuation. Despite the different limitations with each approach, findings were generally consistent across the approaches. Further, the approaches were consistently applied across the study period and thus would not be expected to impact the trends observed in cigarette discontinuation rates over time. Other limitations of this study include that it did not empirically identify *why* cigarette discontinuation rates increased over time more among those who used ENDS than those who did not use ENDS, and we did not evaluate whether trends varied among subgroups of the population such as by age, sex, race/ethnicity, etc.

### Implications

The relationship between ENDS use and cigarette discontinuation among adults who smoke cigarettes in the context of an expanded ENDS marketplace, new tobacco control regulatory actions, and the COVID-19 pandemic, differs from the relationship between ENDS use and cigarette discontinuation among people who smoked cigarettes in 2013/14. Future research can address possible reasons why, such as changes over time in the characteristics of people who smoke cigarettes and use ENDS and/or changes over time in the characteristics of the ENDS products that people use. It is important for public health decisions to be informed by research based on the contemporary ENDS marketplace and circumstances.

## Supplementary material

Supplementary material is available at *Nicotine and Tobacco Research* online.

ntae027_suppl_Supplementary_Material

## Data Availability

Data from the PATH Study may be obtained from a third party and are not publicly available (https://www.icpsr.umich.edu/web/NAHDAP/studies/36231). Application instructions and conditions of use are available at the website above.
