# Supplementary material for: Divergence in Cigarette Discontinuation Rates by Use of Electronic Nicotine Delivery Systems (ENDS): Longitudinal Findings From the United States PATH Study Waves 1–6
Source: Nicotine Tob Res. 2024 Apr 3;27(2):236–43. doi: 10.1093/ntr/ntae027 (PMC11750739; doi:10.1093/ntr/ntae027)
Supplement: ntae027_suppl_Supplementary_Material [file ntae027_suppl_supplementary_material.docx]

Supplemental Figure caption. Cigarette discontinuation defined as P30D smoking at baseline wave and no P30D smoking at follow-up wave for each biennial wave pair; ENDS use defined as P30D ENDS use at follow-up vs. no P30D ENDS use at follow-up; analyses were weighted using the weights appropriate and available for each biennial wave pair as described in detail by Kasza et al. (2022)^17^, including full-sample and 100 replicate weights (i.e., W1-W3 estimates were weighted using W3 all-waves weights for the W1 Cohort, W2-W4 estimates were weighted using W4 all-waves weights for the W1 Cohort, W4-W5 estimates were weighted using W5 single-wave weights for the W4 Cohort, and W5-W6 estimates were weighted using W6 all-waves weights for the W4 Cohort) such that all those who were eligible to participate in any interview pair were included in analyses and estimates represent cigarette discontinuation rates in the population at the time of the follow-up interview for those who were in the CNP at the time of W1 or W4. Sensitivity analyses using only the subset of respondents present in all five or six waves from the W1 cohort depending on which wave pair was involved (i.e., using the W5 all-waves weights for the W1 cohort to evaluate the W4-W5 wave pair, and using the W6 all-waves weights for the W1 cohort to evaluate the W5-W6 wave pair) yielded findings consistent with those reported here.

| **Supplemental Table. Trends in biennial cigarette discontinuation rates between 2013/14 and 2021 (W1 and W6), stratified by P30D ENDS use at follow-up.** | | | | | |
| --- | --- | --- | --- | --- | --- |
|  | **2013/14-2021 (W1-W6)*** | | | | |
|  | Interactions | |  | Stratified | |
| **ENDS use at follow-up** | OR (95%CI) | p |  | OR (95%CI) | p |
| Linear time term |  |  |  |  |  |
| P30D ENDS use | 0.76 (0.57-1.02) | 0.064 |  | 0.83 (0.63, 1.09) | 0.183 |
| No P30D ENDS use |  |  |  | 1.08 (0.96, 1.22) | 0.206 |
| Nonlinear (quadratic) time term | | | | | |
| P30D ENDS use | 1.16 (1.06-1.26) | 0.001 |  | 1.19 (1.09, 1.29) | <.001 |
| No P30D ENDS use |  |  |  | 1.03 (0.99, 1.07) | 0.201 |
| Nonlinear (categorical) time term: 2018/19-2021 vs. 2016/17-2018/19 (W5-W6 vs. W4-W5) | | | | | |
| P30D ENDS use | 1.66 (1.33-2.07) | <.001 |  | 2.12 (1.71-2.64) | <.001 |
| No P30D ENDS use |  |  |  | 1.29 (1.12-1.49) | 0.001 |
| Nonlinear (categorical) time term: 2018/19-2021 vs. 2013/14-2015/16 (W5-W6 vs. W1-W3) | | | | | |
| P30D ENDS use | 1.69 (1.28-2.22) | <.001 |  | 2.73 (2.12-3.53) | <.001 |
| No P30D ENDS use |  |  |  | 1.64 (1.47-1.83) | <.001 |
| Supplemental Table notes. W=Wave; P30D=Past 30-day; 95%CI=95% Confidence Interval. *There were protocol differences between W6 and W1-W5. P30D ENDS number of observations = 4,221 and number of individuals = 2,689; No P30D ENDS use number of observations = 17,531 and number of individuals = 6,633; Analyses were weighted using the W6 all-waves weights for the W1 cohort. | | | | | |
